# Pulmonary Manifestations in Patients With Rheumatoid Arthritis Presenting With Respiratory Symptoms at a Tertiary Care Center in Central India: A Cross-Sectional Study

**DOI:** 10.7759/cureus.117359

**Published:** 2026-10-02

**Authors:** Samruddhi Sanjay, Nalini Humaney, Salil Ganu, Fahad Idrees Shaikh

**Affiliations:** 1 Medical Education, NKP Salve Institute of Medical Sciences and Research Centre and Lata Mangeshkar Hospital, Nagpur, IND; 2 Medicine, NKP Salve Institute of Medical Sciences and Research Centre and Lata Mangeshkar Hospital, Nagpur, IND; 3 Rheumatology, NKP Salve Institute of Medical Sciences and Research Centre and Lata Mangeshkar Hospital, Nagpur, IND

**Keywords:** disease activity score-28, high-resolution computed tomography, interstitial lung disease, pulmonary function test, rheumatoid arthritis, six-minute walk test

## Abstract

Background: Rheumatoid arthritis (RA) is a chronic systemic inflammatory disease that, beyond its classical articular manifestations, frequently involves the lungs. Pulmonary complications, including interstitial lung disease (ILD), pleural disease, airway disease, and pulmonary vascular involvement, are important extra-articular manifestations that contribute substantially to disease morbidity. Many affected patients, however, remain clinically asymptomatic despite radiological or functional abnormalities.

Objectives: The primary objective was to characterize the clinical, functional, and radiological pulmonary findings among patients with RA presenting with respiratory symptoms. The secondary objective was to assess the association of pulmonary function test (PFT) and high-resolution computed tomography (HRCT) abnormalities with disease duration and six-minute walk test (6MWT) distance.

Methods: This cross-sectional observational study was conducted in the Department of General Medicine at a tertiary care, research, and teaching institute over two years (August 2024 to August 2026). Sixty-five consecutive patients aged >18 years with RA diagnosed according to the 2010 American College of Rheumatology/European Alliance of Associations for Rheumatology (ACR/EULAR) classification criteria and presenting with respiratory symptoms were enrolled by convenience sampling after excluding patients with pre-existing pulmonary disease or a connective tissue disorder other than RA. All patients underwent hematological and serological investigations, a 6MWT, spirometry, chest radiography, and HRCT of the chest. Disease activity was assessed using the Disease Activity Score-28 (DAS-28). Unadjusted associations were assessed using the chi-square test, with p < 0.05 considered statistically significant.

Results: Females constituted 72.3% (47/65) of the cohort, and the 41-60-year age group accounted for more than half of the participants (35/65, 53.8%). The mean duration of RA at presentation was 7.1 ± 3.79 years. Chronic cough (43/65, 66.2%) and exertional dyspnea (30/65, 46.1%) were the most common respiratory symptoms, although 46% of patients (30/65) had no abnormal findings on chest auscultation. A restrictive pattern was the most common PFT abnormality (33/65, 50.8%), while a usual interstitial pneumonia (UIP) pattern was the most common abnormal HRCT finding (14/65, 21.5%). Longer disease duration was significantly associated with abnormal PFT findings (χ² = 7.98, df = 3, p = 0.047) and abnormal HRCT findings (χ² = 9.89, df = 3, p = 0.02). Shorter 6MWT distance was significantly associated with abnormal PFT findings (χ² = 20.43, df = 2, p < 0.001) and abnormal HRCT findings (χ² = 10.95, df = 2, p = 0.004).

Conclusion: In this single-center sample of patients with RA presenting with respiratory symptoms, PFT and HRCT abnormalities were common, and longer disease duration and shorter 6MWT distance were associated with abnormal PFT and HRCT findings. These unadjusted cross-sectional associations do not establish causation or predictive value, and the findings cannot be used to estimate the prevalence of pulmonary involvement among all patients with RA. Prospective studies with adjustment for potential confounders are needed.

## Introduction

Rheumatoid arthritis (RA) is a systemic inflammatory disorder of unknown etiology, primarily characterized by peripheral symmetric polyarthritis, with an estimated prevalence of 0.5%-2% in the general population [1]. Extra-articular manifestations occur in 10%-67% of patients across various series [2] and most commonly involve the lungs, presenting as interstitial pneumonitis and fibrosis, pleural disease, pulmonary nodules, organizing pneumonia, pulmonary hypertension, or airway disease affecting both the small and large airways [3,4].

The molecular pathogenesis of RA involves a sequence of abnormal antigen presentation driven by genetic factors, including HLA-DRB1 alleles and the shared epitope, and environmental triggers such as smoking, which promote the citrullination of self-proteins [5]. The resulting neoantigens are recognized by antigen-presenting cells, leading to CD4+ T-cell activation, release of pro-inflammatory cytokines such as TNF-α, IL-1, and IL-6, and production of autoantibodies, including rheumatoid factor (RF) and anti-citrullinated protein antibodies (ACPA/anti-CCP) [6]. Immune complex formation and complement activation stimulate synovial fibroblast proliferation and pannus formation, while matrix metalloproteinase release and RANKL-mediated osteoclast activation contribute to cartilage damage and bone erosion [5,6].

Among the extra-articular complications, interstitial lung disease (ILD) is considered one of the more severe manifestations, and patients with RA are over nine times more likely to develop ILD than the general population [7]. RA-associated ILD (RA-ILD) usually develops within the first five years after the onset of joint symptoms, but in nearly one-fifth of patients, it may precede the articular manifestations, underscoring the importance of early recognition [7,8]. The reported prevalence of RA-ILD ranges from below 1% to nearly 40%, largely reflecting differences in the diagnostic modality used, with detection rates considerably higher when HRCT is used rather than clinical assessment or chest radiography [9]. When both clinically apparent and subclinical disease are considered, more than 40% of patients with RA are estimated to have some form of ILD, although the majority do not have clinically apparent disease, highlighting the potential importance of systematic screening [10].

Pulmonary involvement in RA can affect virtually every compartment of the respiratory system. Parenchymal disease may manifest as usual interstitial pneumonia (UIP) or non-specific interstitial pneumonia (NSIP), organizing pneumonia, or, in severe cases, diffuse alveolar damage. Airway disease may present as upper airway obstruction due to cricoarytenoid joint involvement or as lower airway disease, including bronchiectasis and bronchiolitis. Pleural involvement ranges from asymptomatic pleuritis to pneumothorax and bronchopleural fistula, while pulmonary vascular involvement may manifest as pulmonary hypertension, venous thromboembolism, or pulmonary hemorrhage [1,4]. Pulmonary involvement has long been recognized as a complication of RA, and reports have also documented increased susceptibility to respiratory infections in the presence of these structural abnormalities. Accurate estimation of prevalence, however, remains difficult because of differences in case selection and unmeasured confounders, such as smoking and occupational exposure, across published series [3,4].

Clinically, patients with RA-ILD often present with progressive exertional dyspnea, nonproductive cough, and bibasal crepitations, which may mimic idiopathic pulmonary fibrosis, whereas airway disease may present with hoarseness, recurrent respiratory infections, sputum production, or wheezing. Pleural disease may present with pleuritic chest pain or dullness due to an effusion [11]. The diagnostic workup typically combines serological testing, including RF and anti-CCP, inflammatory markers such as ESR and CRP, and a complete blood count. Pulmonary function testing (PFT) is used to characterize restrictive or obstructive physiology and may detect an early reduction in diffusing capacity for carbon monoxide (DLCO) [12]. Radiologically, chest radiography is comparatively insensitive, whereas HRCT of the chest is considered the gold standard for characterizing patterns such as UIP and NSIP, as well as bronchiectasis, bronchiolitis, pleural abnormalities, and pulmonary nodules [9].

Because pulmonary complications are important contributors to morbidity and mortality in RA [13], and a substantial proportion of affected patients are asymptomatic, systematic clinical, functional, and radiological evaluation in a tertiary care setting may help define patterns of pulmonary involvement within a defined population and facilitate earlier diagnosis, timely management, and risk stratification. This study was therefore undertaken with the primary objective of characterizing the clinical, functional, and radiological pulmonary findings among patients with RA presenting with respiratory symptoms at a tertiary care center, and the secondary objective of assessing the association of PFT and high-resolution computed tomography (HRCT) abnormalities with disease duration and six-minute walk test (6MWT) distance.

## Materials and methods

Study design and setting

This cross-sectional observational study was conducted in the Department of General Medicine at NKP Salve Institute of Medical Sciences and Research Centre and Lata Mangeshkar Hospital, Nagpur, Maharashtra, India. The study was conducted over a period of two years, from August 15, 2024, to August 15, 2026, among patients with RA attending the outpatient department (OPD) or admitted to the inpatient department (IPD).

Sample size calculation

The sample size was calculated to estimate a proportion with acceptable precision, using an expected proportion of pulmonary manifestations of 38% among patients with RA in a tertiary care hospital, based on an earlier study conducted in a similar setting by Gauri et al. [14]. An absolute precision of 12% and a 95% confidence level were used. The sample size was calculated using the following formula:

$n = \frac{Z^2_{1-\alpha/2} \, p(1-p)}{d^2}$

where Z(1-α/2) is the standard normal deviate corresponding to a 5% two-sided alpha error (1.96), p is the expected proportion (0.38), and d is the absolute precision (0.12). The calculated minimum sample size was 63, which was rounded up to 65. Sixty-five participants were ultimately enrolled by convenience sampling, with all consecutive patients who fulfilled the selection criteria during the study period included in the study. Because the study population comprised patients with RA presenting with respiratory symptoms, the proportions reported describe this selected cohort and should not be interpreted as estimates of the prevalence of pulmonary involvement among all patients with RA.

Participants

Inclusion Criteria

All patients aged >18 years who had been diagnosed with RA by a rheumatologist according to the 2010 ACR/EULAR classification criteria [15] and who presented with respiratory symptoms, including dyspnea, cough (productive or nonproductive), or wheezing, were eligible for inclusion. Patients attending the OPD or admitted to the IPD during the study period were included.

Exclusion Criteria

Patients with pre-existing pulmonary diseases, including chronic obstructive pulmonary disease, bronchial asthma, tuberculosis, pneumonia, or acute upper respiratory tract infection; a connective tissue disorder other than RA; chest or spinal deformities, including kyphosis or scoliosis; use of medications affecting the pulmonary system other than those prescribed for RA; pregnancy or lactation; or unwillingness to participate were excluded from the study.

Data collection

Data on sociodemographic characteristics, disease duration, smoking status, clinical symptoms and signs, and comorbidities were recorded using a structured case record form. Laboratory investigations included a complete blood count performed using a fully automated five-part hematology analyzer; C-reactive protein (CRP) measured by immunoturbidimetric assay; anti-cyclic citrullinated peptide (anti-CCP) antibody measured by enzyme-linked immunosorbent assay or chemiluminescent immunoassay; fasting and postprandial blood glucose measured using an electrochemical glucometer; and RF and erythrocyte sedimentation rate (ESR).

Procedure

All 65 participants underwent a complete blood count, RF, anti-CCP antibody testing, ESR, CRP, and blood glucose estimation, followed by a 6MWT, spirometry (PFT), chest radiography, and HRCT of the chest. To assess disease activity, anteroposterior and lateral radiographs of both hands and wrists were also obtained.

Pulmonary Function Testing

Spirometry was performed using a Spirobank G portable spirometer (MIR) in accordance with the American Thoracic Society/European Respiratory Society (ATS/ERS) standardization recommendations [16]. Forced vital capacity (FVC), forced expiratory volume in one second (FEV1), and the FEV1/FVC ratio were recorded, with FVC and FEV1 expressed as percentages of the predicted values. Spirometric patterns were classified as follows: normal (FEV1/FVC ≥ 0.70 and FVC ≥ 80% predicted); obstructive (FEV1/FVC < 0.70, with FVC ≥ 80% predicted); restrictive (FEV1/FVC ≥ 0.70, with FVC < 80% predicted); and mixed (FEV1/FVC < 0.70, with FVC < 80% predicted). Any pattern other than normal was classified as an abnormal PFT. Because lung volumes and diffusing capacity were not measured, a restrictive pattern on spirometry was considered suggestive rather than confirmatory of restriction.

Six-Minute Walk Test

The 6MWT was performed according to the ATS guidelines [17] along a flat, enclosed 30-m corridor under the supervision of a trained observer. Patients were instructed to walk as far as possible within six minutes at their own pace and were permitted to slow down, stop, and rest if necessary. Standardized encouragement was provided at one-minute intervals. The total distance walked was recorded in meters and categorized as >450 m, 350-450 m, or <350 m.

Chest Radiography and HRCT

Posteroanterior chest radiographs were obtained using a Wipro GE Healthcare Tejas XR-6000 unit. HRCT of the chest was performed in all 65 participants using a 16-slice scanner (Toshiba Activion 16), with thin-section acquisition at full inspiration, and was interpreted by a radiologist. Interstitial patterns were classified as UIP, NSIP, or organizing pneumonia according to the ATS/ERS multidisciplinary classification [18]. Other findings, including pleural effusion, bronchiectasis, bronchiolitis, and pulmonary nodules, were recorded using standard descriptive terminology. HRCT was classified as abnormal if any parenchymal, airway, or pleural abnormality was present and as normal otherwise.

Assessment Using Disease Activity Score-28 (DAS-28)

Disease activity was assessed using the Disease Activity Score-28 (DAS28), a composite index originally developed and validated by Prevoo et al. [19] as a modification of the original Disease Activity Score based on a 28-joint count. In this study, DAS-28 was calculated using the ESR (DAS28-ESR), incorporating the 28-tender joint count (TJC28), 28-swollen joint count (SJC28), ESR, and the patient’s global assessment (GH) of disease activity on a 0-100 mm visual analogue scale, using the validated formula:

$\mathrm{DAS28\mathrm{-}ESR} = 0.56 \times \sqrt{\mathrm{TJC}_{28}} + 0.28 \times \sqrt{\mathrm{SJC}_{28}} + 0.70 \times \ln(\mathrm{ESR}) + 0.014 \times \mathrm{GH}$

where TJC28 and SJC28 represent the numbers of tender and swollen joints, respectively, among the 28 joints assessed (shoulders, elbows, wrists, metacarpophalangeal joints, proximal interphalangeal joints, and knees, bilaterally); ESR is expressed in mm/h; and GH represents the patient’s self-rated global assessment of disease activity on a scale of 0 to 100 (0 = no disease activity, 100 = maximal disease activity). Based on the calculated score, disease activity was categorized using the cutoff values validated by van Gestel et al. [20] as follows: remission (DAS-28 <2.6), low disease activity (DAS-28 2.6-3.2), moderate disease activity (DAS-28 >3.2-5.1), and high disease activity (DAS-28 >5.1).

Outcome measures

The primary outcome measure was the pattern and frequency of pulmonary abnormalities, including symptoms, signs, PFT findings, chest radiographic findings, and HRCT findings, among patients with RA presenting with respiratory symptoms. The secondary outcome measures were the associations of PFT and HRCT abnormalities with disease duration and 6MWT distance. Smoking status, serological and inflammatory markers, and DAS-28 disease activity were described but were not formally assessed for their associations with PFT or HRCT abnormalities.

Statistical analysis

Data were entered into Microsoft Excel (Microsoft Corporation, Redmond, WA, USA) and analyzed using IBM SPSS Statistics for Windows, Version 20 (Released 2011; IBM Corp., Armonk, New York). Descriptive statistics were expressed as mean ± standard deviation for continuous variables and as frequencies and percentages for categorical variables; percentages describe this cohort and should not be interpreted as prevalence estimates. Associations of PFT abnormality (normal vs abnormal) and HRCT abnormality (normal vs abnormal) with disease duration category and 6MWT distance category were assessed using the Pearson chi-square test. These were unadjusted analyses; no multivariable adjustment for potential confounders, such as smoking, occupational or environmental exposure, or RA medication, was performed. The chi-square assumption of expected cell counts of at least 5 was assessed; when more than 20% of cells had expected counts <5, exact p-values (Monte Carlo test) were additionally computed as a sensitivity analysis. All 65 participants had complete data for the variables analyzed; therefore, no imputation or exclusion for missing data was required. A two-sided p-value < 0.05 was considered statistically significant.

Ethical considerations

The study was conducted after obtaining approval from the Institutional Ethics Committee of NKP Salve Institute of Medical Sciences & Research Centre and Lata Mangeshkar Hospital, Nagpur (IEC Reference No. 35/2024; approval date: August 9, 2024), in accordance with the ethical principles of the Declaration of Helsinki. Written informed consent was obtained from all participants after they were informed about the purpose, procedures, and potential risks and benefits of the study. Participants were also informed of their right to withdraw from the study at any time without consequences. Confidentiality of participant data was maintained throughout the study.

## Results

A total of 65 patients with RA and respiratory manifestations were enrolled in the study.

The sociodemographic and baseline clinical characteristics of the study participants are presented in Table 1.

**Table 1:** Sociodemographic and baseline clinical characteristics of the study participants (N = 65) RA: rheumatoid arthritis.

Characteristic	Category	Frequency (n)	Percentage (%)
Age group (years)	18-30	6	9.2
	31-40	10	15.4
	41-50	19	29.2
	51-60	16	24.6
	61-70	10	15.4
	>70	4	6.2
Gender	Male	18	27.7
	Female	47	72.3
Duration of RA	<1 year	5	8
	1-5 years	17	26
	6-10 years	29	45
	>10 years	14	21
Smoking status	Non-smoker	42	64
	Smoker	23	36
Socioeconomic status	Upper	15	23
	Middle	25	38.4
	Lower	25	38.4
Residence	Urban	45	69
	Rural	20	31

Most participants were middle-aged, with the 41-50-year age group constituting the largest proportion (29.2%). Women markedly outnumbered men (72.3% vs. 27.7%). Nearly half of the patients (45%) had a disease duration of 6-10 years at presentation (mean 7.1 ± 3.79 years), and 36% had a history of smoking. Patients from the lower (39%) and middle (38%) socioeconomic strata together accounted for more than three-fourths of the cohort, while 69% resided in urban areas.

Pulmonary clinical features and respiratory signs at presentation are presented in Table 2.

**Table 2:** Pulmonary clinical features and respiratory signs at presentation (N = 65)

Category	Finding	Frequency (n)	Percentage (%)
Symptoms	Chronic cough	43	66.2
	Dyspnea on exertion	30	46.1
	Sputum production	18	27.7
	Wheeze	7	10.8
Signs on examination	Crepitations	20	31
	Decreased air entry	15	23
	Crepitations with decreased air entry	25	38
	No abnormal signs	30	46

Chronic cough (66.2%) and exertional dyspnea (46.1%) were the most common presenting respiratory symptoms. On examination, a combination of crepitations and decreased air entry was the most common abnormal finding (38%), whereas 46% of patients had no abnormal respiratory findings on auscultation. This indicates that abnormal pulmonary findings may not always be evident on clinical auscultation.

Comorbidities among the study participants are presented in Table 3.

**Table 3:** Comorbidities among the study participants (N = 65)

Comorbidity	Frequency (n)	Percentage (%)
Hypertension	25	38.4
Diabetes mellitus	18	27.7
Hypothyroidism	12	18.5
None	10	15.4

Hypertension (38.4%) and diabetes mellitus (27.7%) were the most common comorbidities, followed by hypothyroidism (18.5%); 15.4% of patients had no comorbid illness.

Extra-pulmonary (rheumatologic) features of the study participants are presented in Table 4.

**Table 4:** Extra-pulmonary (rheumatologic) features (N = 65)

Feature	Frequency (n)	Percentage (%)
Morning stiffness	62	95.4
Symmetric polyarthritis	59	90.8
Anemia	30	46.1
Systemic symptoms (fever, weight loss)	20	30.8
Subcutaneous nodules	14	21.5
Keratoconjunctivitis sicca	4	6.2

Morning stiffness (95.4%) and symmetric polyarthritis (90.8%) were the most frequently observed articular features in the cohort. Anemia was present in 46.1% of patients, and subcutaneous nodules were noted in 21.5%.

Serological and inflammatory markers of the study participants are presented in Table 5.

**Table 5:** Serology and inflammatory markers (N = 65) Mean ± SD values represent the overall mean for all 65 study participants (not restricted to those with a positive/elevated result). Reference ranges are those of the assays used in this study's laboratory and may vary by method/manufacturer. RF: rheumatoid factor, anti-CCP: anti-cyclic citrullinated peptide antibody, ESR: erythrocyte sedimentation rate (Westergren method), CRP: C-reactive protein.

Test	Positive/elevated (n/65)	Percentage (%)	Mean ± SD (all patients)	Reference range
Rheumatoid factor (RF)	55	84.6	110 ± 45 IU/mL	<14 IU/mL
Anti-CCP antibody	50	76.9	142 ± 60 U/mL	<20 U/mL
ESR (>30 mm/hr)	58	89.2	54 ± 18 mm/hr	Male: <15 mm/hr; Female: <20 mm/hr
CRP (elevated)	48	73.8	14 ± 6 mg/L	<6 mg/L

Rheumatoid factor was positive in 84.6% of patients, while anti-CCP antibody was positive in 76.9%. Inflammatory markers were elevated in a large proportion of patients (ESR, 89.2%; CRP, 73.8%), indicating that elevated inflammatory markers were common in the cohort.

PFT, chest radiography, and HRCT chest findings of the study participants are presented in Table 6.

**Table 6:** PFT, chest radiograph, and HRCT chest findings (N = 65) PFT: Pulmonary function test, HRCT: high-resolution computed tomography, UIP: usual interstitial pneumonia, NSIP: non-specific interstitial pneumonia.

Investigation	Finding	Frequency (n)	Percentage (%)
PFT pattern (N = 65)	Normal	22	33.8
	Restrictive	33	50.8
	Obstructive	6	9.2
	Mixed pattern	4	6.2
Chest X-ray (N = 65)	Normal	34	52.3
	Lower zone opacities	18	27.7
	Pleural effusion	5	7.7
	Cardiomegaly	4	6.2
	Lymphadenopathy	2	3.1
	Atelectasis	1	1.5
	Bronchiectasis	1	1.5
HRCT chest (N = 65)	Normal	28	43.1
	UIP pattern	14	21.5
	NSIP pattern	10	15.4
	Pleural effusion	5	7.7
	Bronchiectasis	4	6.2
	Organizing pneumonia	2	3
	Bronchiolitis	1	1.5
	Pulmonary nodules	1	1.5

A restrictive pattern was the most common PFT abnormality (50.8%), while 33.8% of patients had normal spirometry. Chest radiographs were normal in 52.3% of patients, with lower-zone opacities being the most common abnormality (27.7%). HRCT demonstrated a broader range of abnormalities than chest radiography; although 43.1% of scans were normal, UIP (21.5%) and NSIP (15.4%) patterns were the most common interstitial abnormalities identified.

Details of the 6MWT distance and DAS-28 of the study participants are presented in Table 7.

**Table 7:** Six-minute walk test (6MWT) distance and Disease Activity Score-28 (DAS-28) (N = 65)

Category	Group	Frequency (n)	Percentage (%)
6MWT distance	>450 m	28	43
	350-450 m	22	34
	<350 m	15	23
DAS-28 category	Remission	8	12.3
	Low disease activity	20	30.7
	Moderate disease activity	30	46.2
	High disease activity	7	10.8

On the 6MWT, 43% of patients walked more than 450 m, whereas 23% walked less than 350 m. Based on the DAS-28 scoring, moderate disease activity was the most common category (46.2%), followed by low disease activity (30.7%); 12.3% of patients were in remission.

The association between RA duration and PFT abnormality among the study participants is presented in Table 8.

**Table 8:** Association between duration of rheumatoid arthritis (RA) and pulmonary function test (PFT) abnormality Association was tested using the Pearson chi-square test. p < 0.05 is considered statistically significant.

Duration of RA	Normal PFT	Abnormal PFT	Total	Test statistic (χ²)	df	p-value
<1 year	0	5	5			
1-5 years	10	7	17			
6-10 years	8	21	29			
>10 years	4	10	14			
Total	22	43	65	7.97	3	0.04

A significant association was observed between longer RA disease duration and PFT abnormality (χ² = 7.97, df = 3, p = 0.04). None of the patients with a disease duration of <1 year had an abnormal PFT, compared with 21 of 29 (72%) patients with a disease duration of 6-10 years and 10 of 14 (71%) patients with a disease duration of >10 years.

The association between 6MWT distance and PFT abnormality among the study participants is presented in Table 9.

**Table 9:** Association between six-minute walk test (6MWT) distance and pulmonary function test (PFT) abnormality The association was tested using the Pearson chi-square test. p < 0.05 is considered statistically significant.

6MWT distance	Normal PFT	Abnormal PFT	Total	Test statistic (χ²)	df	p-value
<350 m	2	13	15			
350-450 m	2	20	22			
>450 m	18	10	28			
Total	22	43	65	20.43	2	<0.001

A highly significant association was observed between shorter 6MWT distance and PFT abnormality (χ² = 20.43, df = 2, p < 0.001). An abnormal PFT was present in 13 of 15 (87%) patients who walked <350 m and 20 of 22 (91%) patients who walked 350-450 m, compared with 10 of 28 (36%) patients who walked >450 m.

The association between 6MWT distance and HRCT abnormality is presented in Table 10.

**Table 10:** Association between six-minute walk test (6MWT) distance and high-resolution computed tomography (HRCT) abnormality The association was tested using the Pearson chi-square test. p < 0.05 is considered statistically significant.

6MWT distance	Normal HRCT	Abnormal HRCT	Total	Test statistic (χ²)	df	p-value
<350 m	2	13	15			
350-450 m	8	14	22			
>450 m	18	10	28			
Total	28	37	65	10.95	2	0.004

A significant association was observed between shorter 6MWT distance and abnormal HRCT findings (χ² = 10.95, df = 2, p = 0.004). Abnormal HRCT findings were present in 13 of 15 (87%) patients who walked <350 m, compared with 10 of 28 (36%) patients who walked >450 m.

The association between RA duration and HRCT abnormality is presented in Table 11.

**Table 11:** Association between duration of rheumatoid arthritis (RA) and high-resolution computed tomography (HRCT) abnormality The association was tested using the Pearson chi-square test. p < 0.05 is considered statistically significant.

Duration of RA	Normal HRCT	Abnormal HRCT	Total	Test statistic (χ²)	df	p-value
<1 year	5	0	5			
1-5 years	4	13	17			
6-10 years	14	15	29			
>10 years	5	9	14			
Total	28	37	65	9.2	3	0.02

A significant association was also found between longer disease duration and abnormal HRCT (χ² = 9.2, df = 3, p = 0.02): no HRCT abnormality was seen in patients with disease duration <1 year, whereas 13 of 17 (76%) patients with 1-5 years' duration, 15 of 29 (52%) patients with 6-10 years' duration, and 9 of 14 (64%) patients with disease duration >10 years had an abnormal HRCT.

## Discussion

This single-center, cross-sectional study evaluated 65 patients with RA who presented with respiratory symptoms and were recruited by convenience sampling. The proportions therefore describe this symptomatic cohort and should not be interpreted as estimates of the prevalence of pulmonary involvement among all patients with RA. Furthermore, the associations were unadjusted and should not be interpreted as causal.

Age and gender

In this study, the majority of patients belonged to the 41-50-year age group (29.2%), followed by those aged 51-60 years (24.6%), with a marked female predominance (72.3% vs. 27.7%). Banik et al. [21], in a study of 50 patients with RA, similarly reported a female predominance (58%) and identified the 41-50-year age group as the most affected (36%), although the female predominance was less marked than in the present cohort. The similarity in age distribution between the two studies suggests that middle-aged patients constituted a substantial proportion of the studied RA populations; however, differences in study population and sample size should be considered when comparing these findings.

Duration of RA

The mean duration of RA at presentation in this study was 7.1 ± 3.79 years, with the largest proportion of patients (45%) having a disease duration of 6-10 years. Banik et al. [21] reported a longer disease duration overall, with 50.8% of patients having a disease duration of more than 11 years at presentation. Both studies indicate that a substantial proportion of patients with RA and respiratory symptoms had established disease at the time of evaluation.

Smoking exposure

In this cohort, 36% of patients had a history of smoking, comparable with the 40% reported by Iqbal et al. [22]. Smoking is a common and modifiable exposure in RA; however, because smoking status was recorded only as smoker or nonsmoker in our study, its contribution to the observed pulmonary findings could not be assessed.

Clinical features

Chronic cough (66.2%) and exertional dyspnea (46.1%) were the most common respiratory symptoms in this study. Iqbal et al. [22] similarly reported cough (52.3%) and dyspnea (33.1%) as the leading symptoms, although at lower frequencies, while wheezing was less common in the present study (10.8% vs. 20%). Both studies identified chronic cough as the most frequently reported respiratory symptom among patients with RA.

Comorbidities

Hypertension (38.4%) and diabetes mellitus (27.7%) were the most common comorbidities in this study, occurring at higher frequencies than those reported by Yunt and Solomon [23] (25% and 20%, respectively). Both studies demonstrate a substantial burden of cardiometabolic comorbidity among patients with RA. These comorbidities may potentially contribute to reduced exercise capacity; however, this association was not formally evaluated in the present study.

Signs of pulmonary involvement

Nearly half of the patients (46%) in this study had no abnormal findings on respiratory examination, comparable with the 55% reported by Wilsher et al. [24], while crepitations were noted at similar frequencies in the two studies (31% vs. 30%). These findings indicate that abnormal pulmonary findings may not always be evident on clinical examination and that a normal chest examination does not exclude pulmonary involvement.

Six-minute walk test

In this study, 43% of patients walked more than 450 m on the 6MWT, while 23% walked less than 350 m, demonstrating a range of functional capacity within the cohort. Fischer et al. [25] found that oxygen desaturation and dyspnea scores worsened with increasing RA duration, particularly beyond 10 years, while Ganga et al. [26] demonstrated a significant association between reduced 6MWD and pulmonary manifestations (p = 0.01). The 6MWT is a simple measure of functional capacity; however, performance is influenced by multiple factors, including joint pain, deformity, anemia, deconditioning, and cardiometabolic comorbidity, in addition to pulmonary disease.

Socioeconomic status

Patients from the lower (38.4%) and middle (38.4%) socioeconomic strata predominated in this study. Bakım et al. [27] similarly reported a predominance of patients from the lower socioeconomic class (55%). Differences in socioeconomic distribution may reflect variations in healthcare access and patterns of presentation.

Extra-pulmonary features

Morning stiffness (95.4%) and symmetric polyarthritis (90.8%) were the most common articular features in this study, consistent with the findings of Bogale and Feleke [28], although they were reported at higher frequencies in the present cohort. Subcutaneous nodules (21.5%) were also more frequently observed in this study than in the comparator study.

Serology, inflammatory markers, and PFT

RF and anti-CCP positivity (84.6% and 76.9%, respectively) and elevated ESR and CRP (89.2% and 73.8%, respectively) were higher in this study than the corresponding values reported by Dahani et al. [29] (50%, 40%, 45%, and 40%, respectively). Despite these differences in serological and inflammatory markers, the pattern of PFT abnormalities was similar between the two studies, with a restrictive pattern being the most common abnormality in both (50.8% vs. 50%). Because lung volumes and diffusing capacity were not measured, the restrictive pattern observed in the present study represents a spirometric pattern and does not confirm true restrictive ventilatory impairment.

Chest radiograph and HRCT

Normal chest radiographs were observed in 52.3% of patients in this study, whereas HRCT demonstrated interstitial patterns, with UIP (21.5%) and NSIP (15.4%) being the predominant abnormalities. These findings are consistent with the greater sensitivity of HRCT compared with chest radiography for detecting pulmonary abnormalities in patients with RA [3].

Disease Activity Score-28

Moderate disease activity was the most common category in this study (46.2%), broadly comparable with the pattern reported in the Swedish observational cohort by Gülfe et al. [30], in which 39%-46% of patients had moderate disease activity. The remission rate in the present study (12.3%) was lower than the approximately 23% reported in that cohort. Differences in treatment access, disease duration, and use of biologic agents may partly account for this variation, although these factors were not formally evaluated in the present study. The association between DAS-28 category and PFT or HRCT abnormality was not analyzed in the present study and should be examined in future adequately powered studies.

Association of disease duration with PFT and HRCT abnormality

A significant association was observed between longer disease duration and abnormal PFT (χ² = 7.98, df = 3, p = 0.047) and abnormal HRCT findings (χ² = 9.89, df = 3, p = 0.02). Dahani et al. [29] similarly reported a significant association between disease duration and PFT abnormality (p = 0.01). The pattern was not strictly graded: abnormal HRCT findings were present in 76% of patients with a disease duration of 1-5 years, 52% of those with 6-10 years, and 64% of those with more than 10 years. Only five patients had a disease duration of less than one year, none of whom had an abnormal HRCT. These findings suggest that pulmonary abnormalities were less frequent in very early disease than in more established disease; however, the cross-sectional design and small subgroup sizes preclude conclusions regarding temporal progression or causation.

Association of 6MWT distance with PFT and HRCT abnormality

A shorter 6MWT distance was associated with abnormal PFT (χ² = 20.43, df = 2, p < 0.001) and abnormal HRCT findings (χ² = 10.95, df = 2, p = 0.004), consistent with the association reported by Ganga et al. [26]. However, 6MWT distance is a nonspecific measure of functional capacity, and exertional oxygen desaturation, dyspnea scores, and the reason for stopping the test were not recorded in the present study. Therefore, the observed association does not establish that respiratory impairment was responsible for the reduced walking distance. As no predictive model or diagnostic accuracy analysis was performed, 6MWT distance should not be regarded as a validated screening or predictive tool for pulmonary involvement in RA.

Limitations

This study has several limitations. First, participants were patients with RA who presented with respiratory symptoms and were recruited by convenience sampling at a single tertiary care center. This introduces potential selection and referral bias, limits generalizability, and precludes estimation of the prevalence of pulmonary abnormalities in the broader RA population; asymptomatic patients were not evaluated. Second, the sample size was small (65 patients), and several subgroups, including the five patients with a disease duration of <1 year, were very small. Consequently, the analyses had limited statistical power, and some cells had expected counts below five. Third, the cross-sectional design permits assessment of associations but cannot establish temporal or causal relationships. Fourth, the associations were unadjusted. Smoking status was recorded only as smoker or nonsmoker, with pack-years not analyzed, while occupational and environmental exposures and RA medication history, including methotrexate, biologic agents, and glucocorticoids, were not analyzed. These factors may have contributed to residual confounding. Fifth, DAS-28 disease activity, smoking status, and serological and inflammatory markers were described but were not formally assessed for their associations with PFT or HRCT abnormalities. Sixth, PFT was limited to spirometry without measurement of lung volumes or diffusing capacity; the 6MWT did not include exertional oxygen saturation or dyspnea scoring; and interobserver agreement for HRCT pattern classification was not assessed. Finally, no predictive modeling, diagnostic accuracy analysis, or longitudinal follow-up was performed. Therefore, the clinical utility of disease duration or 6MWT distance for selecting patients for pulmonary screening, as well as their relationship with subsequent functional outcomes or prognosis, remains untested.

## Conclusions

In this single-center sample of patients with RA presenting with respiratory symptoms, abnormalities on PFT and HRCT were common. A restrictive spirometric pattern was the most common functional abnormality, while UIP and NSIP were the most common interstitial findings on HRCT. Nearly half of the patients had no abnormal findings on chest auscultation, suggesting that pulmonary abnormalities may not always be detected by clinical examination alone. Longer disease duration and shorter 6MWT distance were associated with abnormal PFT and HRCT findings in unadjusted analyses; however, these associations do not establish causation and should not be interpreted as validated predictors. Larger multicenter studies that include asymptomatic patients, adjust for smoking, occupational and environmental exposures, and medication use, and formally assess diagnostic accuracy are needed to determine whether these variables have a role in guiding screening for pulmonary involvement in RA.
